# Dairy Fat and Cardiovascular Health

**DOI:** 10.3390/foods9060838

**Published:** 2020-06-26

**Authors:** Esther Sendra

**Affiliations:** Departamento de Tecnologia Agroalimentaria, Escuela Politécnica Superior de Orihuela, Universidad Miguel Hernández, Carretera de Beniel, Km. 3.2., 03312 Orihuela-Alicante, Spain; esther.sendra@umh.es; Tel.: +34-966749735

**Keywords:** dairy foods, dairy fats, cardiovascular disease, saturated fats, polar fats, whole diet

## Abstract

Current scientific evidence points to a neutral or positive effect of dairy fats intake on cardiovascular health. After years of controversy, with many guidelines recommending a reduced intake of dairy products, and preferably low or nonfat dairy foods, current knowledge points to the more appropriate recommendation of moderate consumption of full-fat dairy foods within a healthy lifestyle. Fermented dairy products seem to be the best option as a source of nutrients and cardiovascular health benefits. Previous recommendations were based on cholesterol, saturated fat, and caloric contents, in dairy fat, and their potential impact on serum cholesterol, fasting sugar levels, and blood pressure. However, experimental data point to a more complex scenario in which other actors may play major roles: calcium, bioactive lipids and peptides, and even the food-matrix effect from the dairy food side, and human genetics and environmental factors all impact dairy food-related health issues. Furthermore, cardiovascular health does not rely solely on serum cholesterol levels and blood pressure but also on inflammatory biomarkers. At present, little is known on the true mechanisms underlying the cardioprotective mechanism of dairy fats, and further research in needed to elucidate them.

Dietary recommendations on dairy food consumption are among the most controversial of all. Consumers get nutritional advice not only from reliable scientific-based sources but also from social media, influencers, and other ‘’alternative’’ sources, which are not always based on scientifically verified information; they include passionate groups against milk and dairy products. On the other hand, some other sources present dairy products as ‘’superfoods’’, especially those containing probiotics. Consumers are more confused than ever regarding the nutritional and health effects of dairy products. Institutions and governments have been, and some still are, recommending moderate intake of low or nonfat dairy foods and reduced or null intake of full-fat dairy foods [1]. Not all dairy products have the same effects on human health; butter, cream, and ice-cream are least recommended, as well as dairy foods with high salt, sugar, or added fat. Fermented dairy foods are the most recommended. However, what do we know at present about the relationship between dairy fats, cardiovascular health (CVH), and cardiovascular disease (CVD)? If we run a literature search on a scientific database, like Scopus^®^, we will find thousands of papers, over 150 just in the last 24 months, including reviews but mainly experimental studies. What we have learned from them is that scientific evidence agrees that dairy products are either neutral or beneficial to human CVH [2], and that they are highly nutritious foods (rich in proteins, calcium, and vitamin D, among others). Dairy fats have been associated in the past with increased risk of CVD; however, health effects reported for dairy fat intake do not seem to support past assumptions linking potential health effects of dairy fats on single compounds, or a reduced group of compounds (cholesterol, saturated fatty acids SFA, and calories). It is accepted by the scientific community, and well known by consumers, that diet and lifestyle habits are linked with the development/prevention of chronic disease, and genetic and environmental influences are linked as well; these facts seem to be well supported by scientific evidence [3]. Given this scenario, the effect of dairy products intake on health, specifically the intake of dairy fat, still needs deep investigations.

Recently, Lordan et al. [1] reviewed the relationship between dairy consumption and the incidence of cardiovascular health (CVH), as well as cardiometabolic risk factors. After a long period in which institutions set dietary guidelines recommending the reduction of full-fat dairy foods in favor of low-fat or no-fat dairy, present knowledge, based on observational and experimental studies, points to the benefits of full-fat dairy intake. At present, there is a need to provide a more reliable scientific basis on the effect of dairy fats on CVH and cardiometabolic risk factors as well as dietary guidelines in general. New approaches to diet effect on health are moving to a more focused dietary approach than that in specific foods or compounds present in foods, and are also focusing on phenotypic differences among individuals.

Saturated fats (SFA), cholesterol, and caloric content, of dairy products, have been the basis of past argumentation against dairy fats. SFA have been considered to negatively affect CVH, and SFA have been taken as a whole; nowadays, dairy SFA have been proven to have different effects on human health than industrial SFA [4], so they cannot be considered as a single nutrient and need to be evaluated as individual molecules with specific functions [2]. It has been widely accepted that the replacement of SFA with unsaturated fatty (UFA) acids may enhance CVH [5], and many attempts are being made to enhance UFA in milk and dairy foods, such as the enrichment of cheese with n3-PUFA with successful impact on CVD biomarkers [6]. However, there is no clear evidence of the benefits of reducing SFA content in dairy foods on CVH [7]. The same can be said for cholesterol; no clear relationship can be established between dietary cholesterol and serum cholesterol levels—only hyper-responders show increased serum LDL cholesterol as a response to dietary cholesterol. The effect of dairy fat on serum cholesterol has also been proven to be dependent on the dairy food, thus pointing to matrix effects: full fat milk behaves similarly to butter, whereas cheese has the lowest impact on serum cholesterol levels [8]. Butter consumption increases cholesterol levels, however its long term effect on mortality or CV health is unclear. The mechanisms responsible for the lowering of cholesterol concentrations in cheese, compared to butter intake, remain unclear. There is a theory that claims that the combination of high calcium and fatty acid in the intestines after cheese ingestion may favor soap formation and enhance fat excretion [9]. Given this dairy matrix effect, the traditional reductionist view of single nutrients is moving towards a whole-food and diet approach, given that the matrix has implications in digestion, absorption, and metabolism [10].

Low-fat dairy products have been recommended in most dietary guidelines since the 70′s, and low-fat products have been considered as healthy ever since. Since the 70′s, full-fat dairy consumption has decreased, whereas low and nonfat dairy consumption have increased. The food industry has modified processes and formulas (mainly increasing sugar content) to enhance product taste, and palatability, lost due to the reduced fat. As a result, increased intake of low-fat dairy has led to enhanced sugar consumption, and reduced intake of vitamin D, vitamin K, and bioactive lipids. However, full fat has been recently associated with decreased obesity, type II diabetes, and blood pressure, as reviewed by Lordan [1].

Metabolic syndrome, hypertension, type II diabetes, and obesity are conditions connected to CVD. Several studies have evaluated dairy products’ effect on hypertension; evidence points to a positive impact of milk and dairy products on hypertension, probably mediated by bioactive lipids and peptides present in dairy foods [2]. Regarding dairy products’ effect on diabetes, dairy foods have neutral or positive effect on risk reduction when fermented dairy products are consumed [11]. Regarding the protection against diabetes due to dairy, mechanisms are unclear; again, bioactive lipids, and peptides, may be involved, as well as the complexity of the food matrix [1]. High-energy intake is the main factor responsible for obesity and insulin resistance. Conflicting results have been reported on the effect of dairy foods on obesity, with studies reporting inverse relationship between dairy consumption and body mass index and weight gain, and others reporting some weight gain [12].

Anti-inflammatory mechanisms and dairy foods: studies indicate that dairy products may be cardioprotective due to lower levels of inflammatory markers; the involved mechanisms are still to be elucidated, and may include specific fatty acids, and polar lipids (whose bioactivity is higher in fermented milks as well as small ruminant milk), which seem to inhibit platelet-activating factor [1,13]. In 2018, Lordan [1] summarized the findings from observational studies investigating the consumption of dairy foods on inflammatory markers related to CVD, obesity, and metabolic syndrome, in healthy and diseased individuals. Compiled studies were published from 2005 until 2017, in different countries. Overall, ten of the studies reported neutral effect of dairy products on inflammatory markers, whereas in nine of them dairy products caused favorable biochemical changes that provided health benefits.

Trans fatty acids (TFA) have also been associated with CVD. Dietary guidelines recommend limiting TFA ingestion. However, there is increased evidence pointing to differentiated health effect of TFA depending on the food source, suggesting that specific TFA from ruminant source such as trans-vaccenic and rumenic acids may be beneficial against CVD (anti atherosclerosis and anti-inflammatory) [2], as opposed to those of processed fats such elaidic acid, which increase the risk of CVD (atherosclerosis and plaque formation) [14]. Further research is needed to confirm that ruminant TFA may be beneficial to CVH.

Fermented dairy products, including mainly fermented milks (yogurt, kefir, kumis, and others) and cheese, are increasingly popular. Fermented milks are perceived as healthy foods, adding to the nutritional value fermentation metabolites and the delivery of probiotics. Fermented milk intake has been associated with beneficial effects on CVH [15], including reduced risk of diabetes [11] and increased HDL cholesterol [16]. Cheese consumption also seems to have less effect on CVH than expected. Mechanisms behind such observations may be the presence of bioactive peptides (including Angiotensin-converting enzyme inhibitors) and lipids with specific functionality [2] and high calcium content [9]. Additionally, fermentation metabolites such as bioactive molecules, as well as forms of vitamin K, the presence of probiotics and the matrix effect may play a role in CVH protection [1]. Special fermented milks such as kefir are presumed to possess valuable health properties, but their relationship with CVH is still to be explored. It seems clear that fermented dairy foods are more beneficial to CVH than nonfermented dairy, however the true mechanisms involved are still to be unveiled and understood.

In search of healthier dairy foods, the industry has developed special products such as cholesterol-lowering dairy products (mainly fermented milks containing stanols/sterols), which effectively reduce serum cholesterol levels; plan-based milk-alternatives, whose long-term effects on CVH are not known, have a different nutritional profile than milk (calcium, protein, and vitamins constitute the differences). Additionally, small ruminant milk is being promoted as a bovine milk alternative; both caprine and ovine milk are highly beneficial due to their high digestibility, have a more beneficial fatty acid profile (high in UFA, medium chain triglycerides), and have high vitamin and bioactive lipids contents [2,13]. Their higher digestibility allows for better uptake of bioactive lipids. Still, more research is needed on ovine and caprine product effects on CVH.

Current scientific evidence does not support the dietary recommendation to decrease or avoid full-fat dairy foods, as they seem to be beneficial or neutral to CVH. Both CVD and food are complex, and need to be approached as such. Genetics, environmental factors, and lifestyle greatly affect CVH. Previous approaches based on cholesterol, blood pressure, and obesity, such as CVD basic indicators, and cholesterol, SFA, and caloric content of full-fat dairy foods, as negative factors, have proven to be simplistic. New models, including systemic inflammation biomarkers, need to be considered when approaching CVD [1]. Regarding dairy foods, other factors such as matrix-effects, bioactive lipids and peptides, other biomolecules, and vitamins D and K need to be explored to elucidate the cardioprotective mechanisms of dairy products.
